# Supplementary material for: A receptor and neuron that activate a circuit limiting sucrose consumption
Source: eLife. 2017 Mar 23;6:e24992. doi: 10.7554/eLife.24992 (PMC5388533; doi:10.7554/eLife.24992)
Supplement: Supplementary file 1. — (A) cloning primers generated for the CRISPR guide chiRNA, (B) CRISPR donor plasmid, and (C) screening to verify the removal of IR60b coding regions. In (A), lowercase nucleotides denote primer sequences homologous to the Gibson Assembly plasmid regions. DOI: http://dx.doi.org/10.7554/eLife.24992.016 [file elife-24992-supp1.docx]

**Supplementary File 1.**

**(A): CRISPR Guide chiRNA cloning primers**

| Primer | Name | Sequence |
| --- | --- | --- |
| 1 | chiRNA R | gaagtattgaggaaaacata |
| 2 | IR60b 5pchiRNA F | GGCTCTTTACGAAAATCTCGgttttagagctagaaatagc |
| 3 | IR60b 3pchiRNA F | GGCATGGCCATGATAAGCAGgttttagagctagaaatagc |

**Gibson Assembly plasmid sequence indicated as lower case.*

**(B): CRISPR Donor plasmid cloning primers**

| Primer | Name | Sequence | Enzyme |
| --- | --- | --- | --- |
| 4 | IR60bH1F | GCGCCTGAATTCATCCCAAATTGGTTTTCGGCTGC | EcoRI |
| 5 | IR60bH1R | GCGCCTCCGCGGGATTTTCGTAAAGAGCCTGCATGGT | SacII |
| 6 | IR60bH2F | GCGCCTACTAGTCTTATCATGGCCATGCCCTTGC | SpeI |
| 7 | IR60bH2R | GCGCCTCTCGAGATCCTCTAGAGATTTCAGCATA | XhoI |

**(C): Screening primers for IR60b deletion verification**

| Primer | Name | Sequence |
| --- | --- | --- |
| 8 | Screen60bF | CAGCGCTTGTAGACATTCTG |
| 9 | Screen60bH1F | ATCCCAAATTGGTTTTCGGCTGC |
| 10 | Screen60bH2R | CTGAGACTGCCCAGCC |
| 11 | Screen60bR | GCTCCCCTTATCGACTTCAGG |
